# Rewiring ERBB3 and ERK signaling confers resistance to FGFR1 inhibition in gastrointestinal cancer harbored an ERBB3-E928G mutation

**DOI:** 10.1007/s13238-020-00749-z

**Published:** 2020-07-06

**Authors:** Xiang Yang, Hongxiao Wang, Enjun Xie, Biyao Tang, Qingdian Mu, Zijun Song, Junyi Chen, Fudi Wang, Junxia Min

**Affiliations:** The First Affiliated Hospital, Institute of Translational Medicine, Zhejiang University School of Medicine, Hangzhou, 310058 China

**Dear Editor,**

Recently, a large number of studies found that activation of ERBB3 (Erb-B2 receptor tyrosine kinase 3, also known as HER3) may be one of the major mechanisms underlying resistance to therapies that target the EGFR (epidermal growth factor receptor), HER2, and other receptor tyrosine kinases (RTKs) (Chen et al., 2011; Choi et al., 2012; Ross et al., 2018). Interestingly, mutations in ERBB3 are commonly reported in gastrointestinal (GI) cancer, with mutations identified in approximately 12% of stomach and colorectal cancer cases (Jaiswal et al., 2013). Here we also mined TCGA-generated data in the cBioPortal for Cancer Genomics regarding ERBB3 and its related RTKs (ERBB2, EGFR, VEGFR, IGF1R, MET and FGFR) family members) in GI cancer. Our analysis revealed that the *ERBB3* gene is genetically altered in 9% (75 of total 797 queried samples) of GI samples analyzed, making *ERBB3* the third most commonly altered gene after *ERBB2* (15%) and *EGFR* (10%). Interestingly, unlike other RTKs, mutations in *ERBB3* (the green color labeled) accounted for 73.3% of *ERBB3* genetic alterations (55 mutations out of total 75 *ERBB3* genetically altered samples) (Fig. S1), suggesting that mutations in *ERBB3* might play a role in the progression of GI cancer. We therefore hypothesized that ERBB3 might play a central role in conferring resistance to commonly used targeted therapies in patients with GI cancer; moreover, we hypothesized that pharmacologically blocking ERBB3 might help reduce this resistance to tyrosine kinase inhibitors (TKIs), thereby delaying relapse and improving patient outcome. Here we demonstrate that ERBB3-E928G mutated cells are highly resistant to FGFR1 inhibition via an increased activation of ERBB3 and its downstream ERK signaling pathways, and combination of ERBB3 monoclonal antibody LJM716 and the specific FGFR1 inhibitor PD173074 synergistically suppressed the growth of ERBB3-mutated gastrointestinal cancer and potently overcome tumor refractory to FGFR1 inhibitors.

We firstly investigated whether the presence of mutant ERBB3 proteins affects the response to clinically available TKIs in a subset of GI cancer cell lines. Specifically, we measured the effects of TKIs in four GI cancer cell lines carrying representative ERBB3 hotspot mutations, including CW-2 cells (ERBB3-E928G), KYSE150 cells (ERBB3-D297Y), HCT116 cells (ERBB3-Q261*), HCT15 cells (ERBB3-N126K), and AGS control cell line with wildtype (WT) ERBB3 (Fig. 1A). Notably, CW-2 cells have significantly higher levels of phosphorylated ERBB3 (pERBB3) compared to the other four screened GI cancer cell lines (Fig. 1A). For each cell line, we measured cell viability in the presence of various concentrations of Gefitinib (an EGFR inhibitor), Lapatinib (a HER2 inhibitor), Apatinib (a VEGFR-2 inhibitor), Linsitinib (an IGF-1R inhibitor), Tivantinib (a c-Met inhibitor), and BGJ398 (an FGFR1-3 inhibitor). Results showed that only CW-2 cells, which harbor the ERBB3-E928G mutation, were more resistant to the FGFR1-3 inhibitor BGJ398 compared to the other four cell lines (Fig. 1A). Results of the time course experiment validated that CW-2 cells were virtually unaffected by BGJ398 treatment compared with AGS control cells (Fig. 1B). Importantly, two FGFR4 inhibitors, BLU554 (Fig. 1C) and BLU9931 (Fig. S2), had a similar effect on CW-2 cells and AGS cells. Given that BGJ398 inhibits FGFR1, FGFR2 and FGFR3, we examined which FGFR underlies the resistance to BGJ398 in CW-2 cells. As shown in Fig. 1D, CW-2 cells are more resistant to the FGFR1-specific inhibitor PD173074 (Nguyen et al., 2013) compared to AGS cells. We also used RNAi to knock down *FGFR1* expression in both CW-2 and AGS cells (Fig. S3). As shown in Fig. 1E, knocking down *FGFR1* using two different shRNA constructs significantly reduced the viability of AGS cells compared to CW-2 cells. These results suggest that the ERBB3-E928G mutation underlies the cellular resistance to FGFR1 inhibitors. Thus, we hypothesized that the E928G mutation in ERBB3 kinase domain might coordinate with FGFR1 to maintain the growth and survival of CW-2 cells in the presence of FGFR1 inhibition.

**Figure 1:**
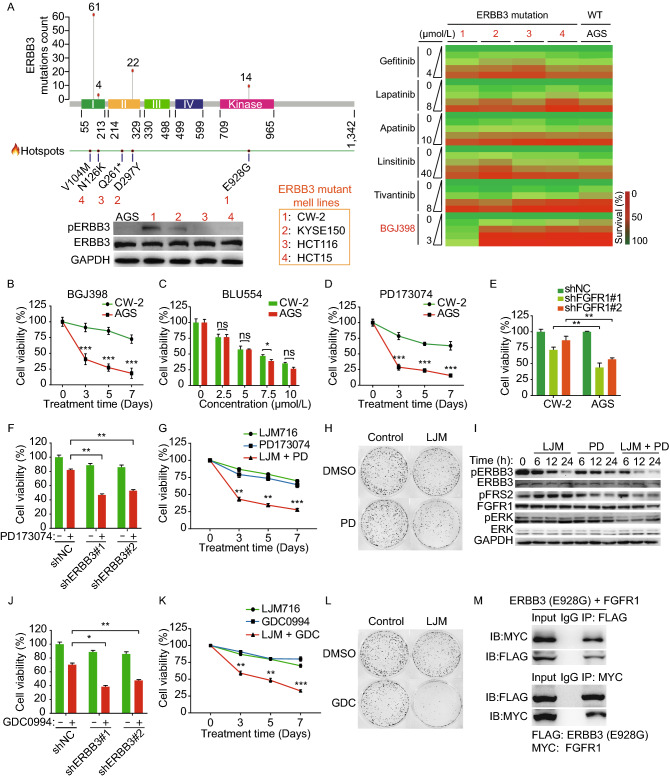
**Identification of ERBB3 kinase domain mutant E928G contributes to resistance of GI cancer cells to FGFR1 inhibition via downstream MEK-ERK signaling pathway.** (A) A functional screen of TKIs sensitivity was performed in 5 selected GI cancer cell lines, which closely recapitulate the spectrum of ERBB3 mutation across 1165 human cancer samples (Cancer Hotspots Database, https://www.cancerhotspots.org). On the top of the left panel: the numbers below the figure refer to amino acid positions, and the hotspot mutation sites are shown as solid red circles. A total of 214 ERBB3 mutations were identified in 1,165 cancer samples, and the three most prevalent hotspots (V104, D297, and E928) are shown, with the number of samples indicated. On the bottom of the left panel: protein levels of pERBB3 and ERBB3 in 5 selected GI cancer cell lines measured by Western blot. GAPDH serves as a loading control. The right panel: heat map shows the screening results of six TKIs in a subset of five separate GI cancer cell lines with various ERBB3 mutations (AGS cells express wild-type ERBB3). Cells were treated with the indicated TKIs at the indicated concentrations, and cell viability was measured after 72 h. (B) CW-2 and AGS cells were treated with 2 μmol/L BGJ398, and cell viability was measured on day 0, 3, 5, and 7. (C) Cell viability of CW-2 and AGS cells treated for 3 days with the FGFR4 inhibitor BLU554 at the indicated concentrations. (D) Cell viability of CW-2 and AGS cells treated with 3 μmol/L PD173074 for 7 days. (E) Cell viability of CW-2 and AGS cells infected with a control shRNA lentivirus construct (shNC) or two different anti-*FGFR1* shRNA constructs. (F) Cell viability was measured in CW-2 cells transfected with the indicated shRNA construct and then treated for 3 days with or without 3 μmol/L PD173074. (G) Time course of cell viability of CW-2 cells treated with either 10 μg/mL LJM716 alone, 3 μmol/L PD173074 alone or both LJM716 and PD173074. (H) Foci formation was measured for CW-2 cells treated for 2 weeks with DMSO (control), 10 μg/mL LJM716, 2 μmol/L BGJ398, or both LJM716 and BGJ398. (I) CW-2 cells were treated with relative high dose of LJM716 (20 μg/mL), PD173074 (10 μmol/L), or both, followed by western blot analyses at the indicated time. (J) Cell viability was measured in CW-2 cells subjected to shRNA-mediated *ERBB3* knockdown followed by 3 days of treatment with 10 μmol/L GDC0994. (K) Time course of cell viability of CW-2 cells treated with either 10 μg/mL LJM716 alone, 10 μmol/L GDC0994 alone or both LJM716 and GDC0994. (L) 2 weeks foci formation was measured for CW-2 cells treated with DMSO (control), 10 μg/mL LJM716, 10 μmol/L GDC0994, or both LJM716 and BGJ398. (M) HEK293T cells were co-transfected with FLAG-tagged ERBB3-E928G and MYC-tagged FGFR1, 3 days after transfection, cell lysates were subjected to Co-IP using anti-FLAG and anti-MYC antibodies. *P < 0.05, **P < 0.01, ***P < 0.001, and ns, not significant

In order to investigate whether the resistance of CW-2 cells to FGFR1 inhibition depends on its increased ERBB3 activation, we knocked down endogenous *ERBB3* in CW-2 cells using two different lentiviral shRNAs that specifically target *ERBB3*, thereby reducing both total ERBB3 and pERBB3 levels (Fig. S4). We found that knocking down ERBB3 significantly increased the response to PD173074 (Fig. 1F). LJM716 is an ERBB3-neutralizing antibody that inhibits both ligand-induced and ligand-independent ERBB3 activation (Garrett et al., 2013). Therefore, we tested the effect of combining LJM716 with the FGFR1 inhibitor PD173074 on cell viability in CW-2 cells. Compared with either treatment alone, treatment with both LJM716 and PD173074 potently reduced both cell viability through 7 days (Fig. 1G) and foci formation (Fig. 1H). Furthermore, western blot results showed that the cleaved-PARP and cleaved-caspase-3 levels are drastically increased in LJM716 and PD173074 combination treatment group, which suggests that the combination treatment induces potent apoptosis in CW-2 cells (Fig. S5). Taken together, these results suggest that blocking activation of the ERBB3-E298G mutant restores the cell’s sensitivity to FGFR1 inhibitor.

Based on these findings, we tested whether co-treating CW-2 cells with LJM716 together with PD173074 affects downstream signaling of ERBB3 and FGFR1 pathways, such as MEK/ERK, PI3K/AKT and/or pFRS2 pathways. To test these signaling related protein levels, CW-2 cells were treated with each drug at indicated time points. We found that pFRS2 was significantly decreased by the FGFR1 inhibitor PD173074, but was unaffected by LJM716 (Fig. 1I). Notably, both pERBB3 and pERK were significantly decreased upon co-treatment with LJM716 and PD173074 (Fig. 1I). In addition, co-treatment with LJM716 and PD173074 had no significant effect on both pAKT-T308 (pAKT-T) and pAKT-S473 (pAKT-S), pJNK, or phospho-p38 (p-p38) level (Fig. S6). To further detect the canonical downstream ERBB3 and FGFR1 signaling pathways, we measured the protein levels of pFRS2, pAKT and pERK. We found that the pAKT-T and the pERK levels were significantly higher in CW-2 cells compared to those in AGS WT control cells (Fig. S7). Notably, the cell viability assay showed that CW-2 cells are more resistant to ERK inhibitor GDC0994 compared with AGS cells (Fig. S8A). By contrast, the PI3K inhibitor LY294002 showed no significant difference between these two cell lines (Fig. S8B). Taken together, these data indicate that ERK activation—but not AKT signaling—plays a role in the resistance of CW-2 cells to FGFR1 inhibition, and targeting both ERBB3 and FGFR1 has a synergistic effect in terms of inhibiting both ERBB3 and ERK signaling pathways.

Next, we tested the sensitivity of CW-2 cells to either the ERK inhibitor GDC0994 or the PI3K inhibitor LY294002 following shRNA-mediated knockdown of ERBB3. We found that knocking down *ERBB3* restored the sensitivity of CW-2 cells to GDC00994 (Fig. 1J) but did not affect sensitivity to LY294002 (Fig. S9). To further test whether the ERK pathway plays a role in CW-2 cells, we measured the viability and foci formation of cells co-treated with the anti-ERBB3 antibody LJM716 and the ERK inhibitor GDC0994. We found that inhibiting both ERBB3 and ERK significantly decreased cell viability (Fig. 1K) and reduced foci formation (Fig. 1L). Similar results were obtained when CW-2 cells were co-treated with LJM716 and the MEK1/2 inhibitor Trametinib (Fig. S10).

A growing body of evidence supports the notion that mutations in ERBB3 confer resistance to various TKIs, although the underlying molecular mechanisms are unknown. Previous studies suggested that mutant forms of ERBB3 can be activated by forming heterodimers with RTKs from the same family such as HER2 or EGFR (Sithanandam and Anderson, 2008; Jaiswal et al., 2013) and even forming heterodimers with RTKs from the different families such as IGF1-R and c-MET (Huang et al., 2010; Tanizaki et al., 2011). We next performed a co-immunoprecipitation (Co-IP) assay using lysates prepared from HEK293T cells co-transfected with tagged ERBB3-E928G and FGFR1 constructs. Consistent with our hypothesis, E928G mutant ERBB3 co-precipitated with FGFR1 (Fig. 1M). We also performed Co-IP to measure endogenous interaction of ERBB3 and FGFR1 in CW-2 and AGS cells, respectively. Results showed that E928G mutant ERBB3 interacts with FGFR1 in CW-2 cells, whereas WT ERBB3 did not interact with FGFR1 in AGS cells (Fig. S11). Taken together, these data support the notion that the ERBB3-E928G mutant interacts with FGFR1 in order to activate downstream MEK/ERK signaling.

Lastly, we examined the *in vivo* effect of targeting both ERBB3 and FGFR1. CW-2 subcutaneously tumor-bearing murine model was generated and treated with LJM716, PD173076, or both. Compared to control-treated mice and mice treated with either LJM716 or PD173074 alone, the mice treated with both LJM716 and PD173074 had significantly less tumor growth (Fig. 2A–C). In addition—and consistent with our *in vitro* results—immunohistochemistry (IHC) of the xenograft tumors revealed significantly lower levels of both pERBB3 (Fig. 2D) and pERK (Fig. 2E) staining in the co-treated tumor xenografts compared to the other three groups. Next, we measured cell proliferation and apoptosis in the tumors using Ki-67 and TUNEL staining, respectively. We found that cell proliferation was significantly reduced (Fig. 2F) and apoptosis was significantly increased (Fig. 2G) in the co-treated tumor xenografts compared to the other three groups. Taken together, these *in vivo* data provide compelling evidence that blocking both ERBB3 and FGFR1 may be a viable strategy for treating GI cancer harboring the ERBB3-E928G mutation.

**Figure 2:**
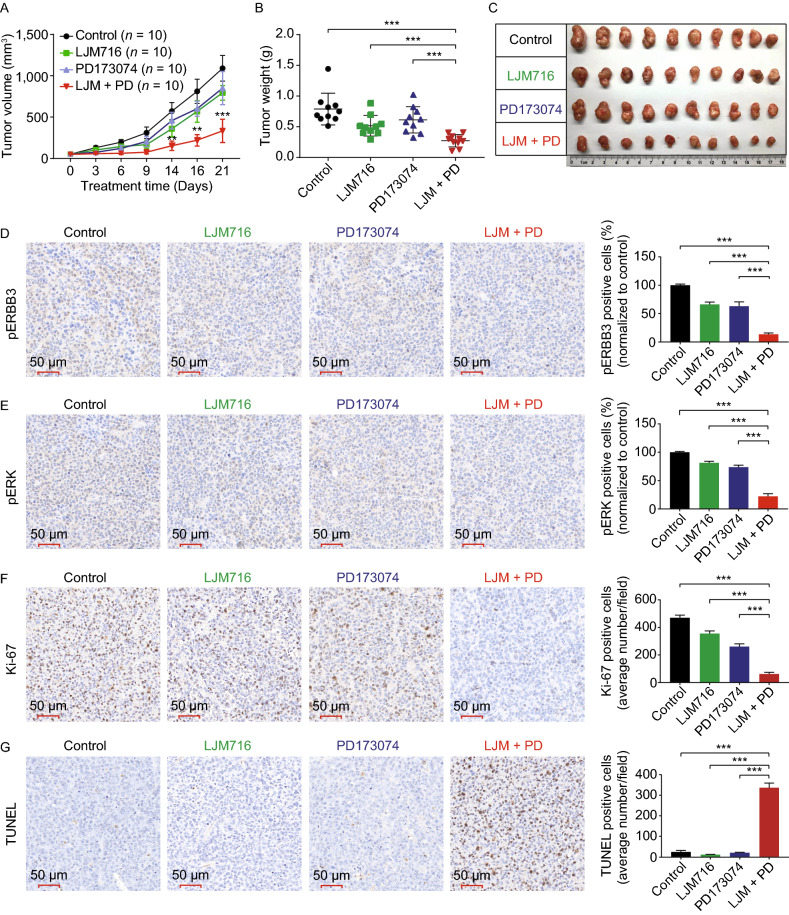
***In vivo*** **analysis of blocking both ERBB3 and FGFR1 in a CW-2 xenograft mouse model.** (A) Female BALB/c nude mice were injected subcutaneously with 5 × 10^6^ CW-2 cells per site and then randomly assigned to receive intraperitoneal injections of vehicle (control), LJM716 (25 mg/kg body weight), PD173074 (20 mg/kg body weight), or both LJM716 and PD173074 every 2 days for a total of 3 weeks; tumor volume was measured at the indicated time (n refers to the number of tumors in each treatment group). (B) Summary of tumor weight measured on day 21. (C) Photographs of all 10 tumors in the indicated treatment groups. (D–G) On day 21, the tumors were sectioned and analyzed using pERBB3 IHC (D), pERK IHC (E), Ki-67 staining (F), and TUNEL staining (G). The graphs at the right show the summary data measured from 3 tumors per group. *P < 0.05, **P < 0.01, ***P < 0.001, and ns, not significant

In conclusion, we functionally screened the sensitivity of GI cancer cell lines harboring various mutations in ERBB3 to clinically available receptor tyrosine kinase inhibitors. We found that cells, which carry the hotspot mutation E928G in the kinase domain (Verlingue et al., 2018), are highly resistant to treatment with FGFR1 inhibition. With respect to the underlying mechanism, we found that these cells require either FGFR1 or ERK activation for their growth and survival. We provide compelling evidence supporting the notion that combined treatment with both ERBB3 and FGFR1 inhibitors may provide an effective approach for clinical benefits of GI cancer harboring mutations in the kinase domain of ERBB3, which might improve the survival and outcome of those patients. Future studies are therefore warranted in order to test the feasibility and efficacy of using this combination therapy to treat gastrointestinal cancer.

## Electronic supplementary material

Below is the link to the electronic supplementary material.

Supplementary file1 (PDF 724 kb)
