# Supplementary material for: Rewiring ERBB3 and ERK signaling confers resistance to FGFR1 inhibition in gastrointestinal cancer harbored an ERBB3-E928G mutation
Source: Protein Cell. 2020 Jul 6;11(12):915–20. doi: 10.1007/s13238-020-00749-z (PMC7719122; doi:10.1007/s13238-020-00749-z)
Supplement: Supplementary file 1 — Supplementary file1 (PDF 724 kb) [file 13238_2020_749_MOESM1_ESM.pdf]

Supplementary Materials

Rewiring ERBB3 and ERK Signaling Confers Resistance to FGFR1 Inhibition in Gastrointestinal Cancer Harbored an ERBB3-E928G Mutation

Xiang Yang#, Hongxiao Wang#, Enjun Xie, Biyao Tang, Qingdian Mu, Zijun Song, Junyi Chen, Fudi Wang, and Junxia Min*

The First Affiliated Hospital, Institute of Translational Medicine, School of Public Health, Zhejiang University School of Medicine, Hangzhou 310058, China

# These authors contributed equally to this work.

* **Corresponding Author**: Junxia Min, The First Affiliated Hospital, Institute of Translational Medicine, Zhejiang University School of Medicine, Hangzhou 310058, China, Phone: +086-17767060111, Email: junxiamin@zju.edu.cn

Supplementary Figure

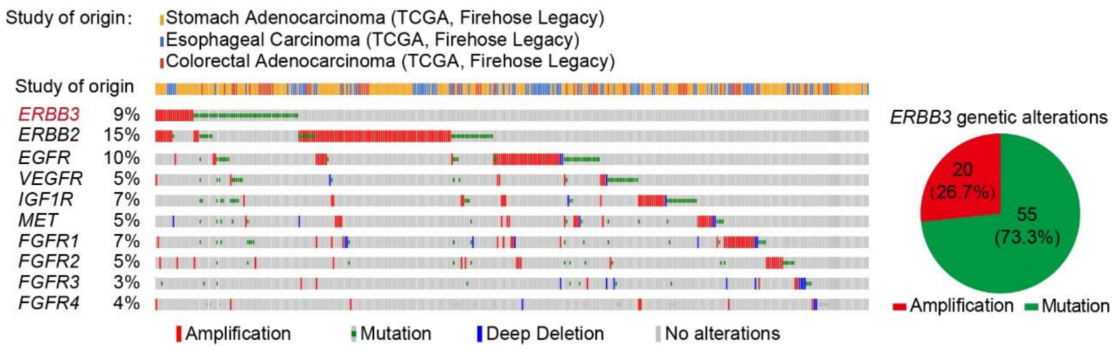

Figure S1: "Oncoprint" results based on our analysis of data mined from the cBioPortal for Cancer Genomics. The left panel shows genetic alterations in ERBB3 and other related RTKs analyzed in 3 studies from TCGA database, including stomach adenocarcinoma, esophageal carcinoma, and colorectal adenocarcinoma. The pie chart shows the sample size (percentage) of *ERBB3* genetic alterations.

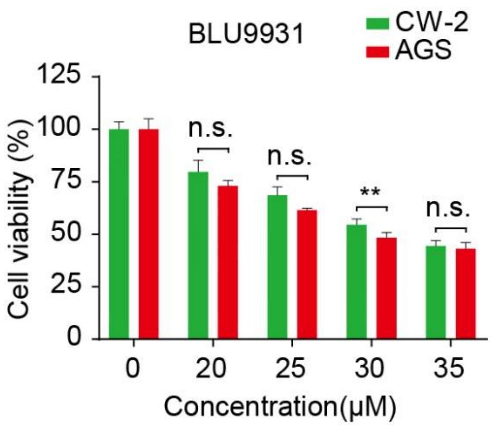

Figure S2: FGFR4 inhibitor BLU9931 had a similar effect on CW-2 cells and AGS cells. Cell viability of CW-2 and AGS cells treated with the indicated concentrations of BLU9931. * $P < 0.05$, ** $P < 0.01$, *** $P < 0.001$, and n.s., not significant.

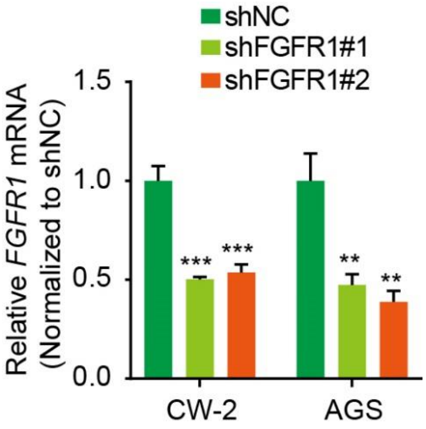

Figure S3: FGFR1 knocking down efficiency in CW-2 and AGS cells. *FGFR1* mRNA was measured in CW-2 and AGS cells transfected with either a control shRNA or two shRNA constructs that target *FGFR1* using real-time PCR analysis and was normalized to the control. * $P < 0.05$, ** $P < 0.01$, *** $P < 0.001$, and n.s., not significant.

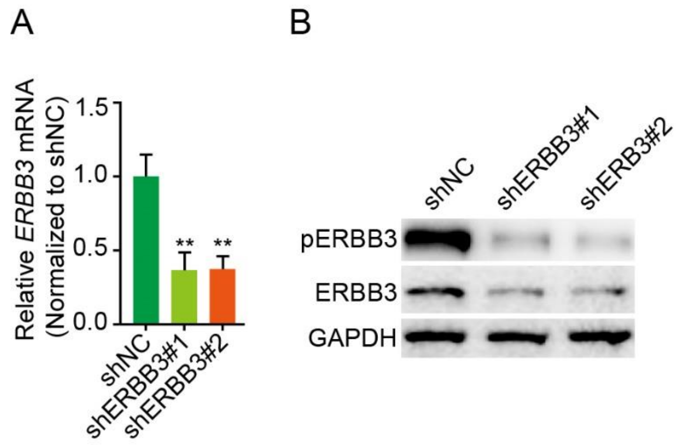

Figure S4: ERBB3 knocking down efficiency in CW-2 cells. (A) *ERBB3* mRNA was measured in CW-2 cells transfected with either a control shRNA or two shRNA constructs that target *ERBB3*. (B) pERBB3 and total ERBB3 in CW-2 cells were tested three days after transfection with the indicated shRNA constructs. * $P < 0.05$, ** $P < 0.01$, *** $P < 0.001$, and n.s., not significant.

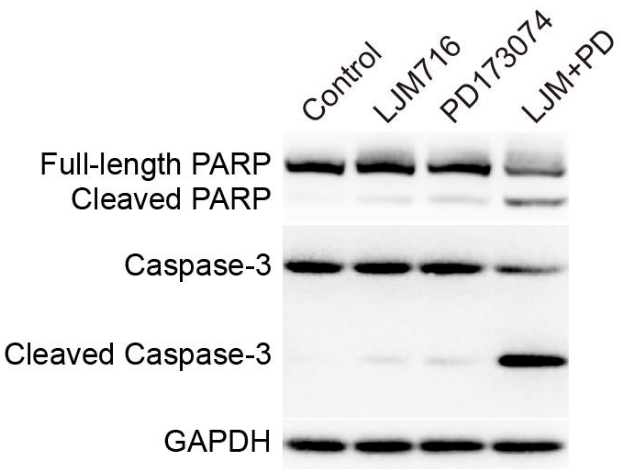

Figure S5: LJM716 and PD173074 co-treatment induced potent apoptosis in CW-2 cells. CW-2 cells were treated with or without 20 µg/ml of LJM716, 10 µM of PD173074 or both for 48h. The apoptosis markers (cleaved-PARP and cleaved-Caspase-3) were tested through western blot analyses. GAPDH serves as a loading control.

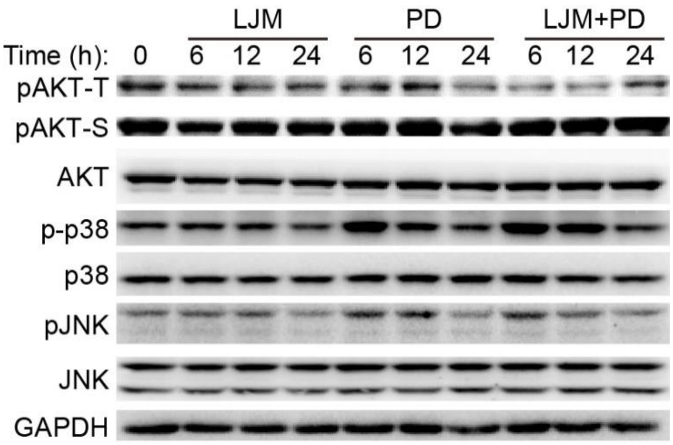

Figure S6: LJM716 and PD173074 co-treatment had no significant effect on pAKT-T, pAKT-S, pJNK, or p-p38 protein levels. CW-2 cells were treated with LJM716 (20 µg/ml), PD173074 (10 µM), or both, followed by western blot analyses at the indicated time.

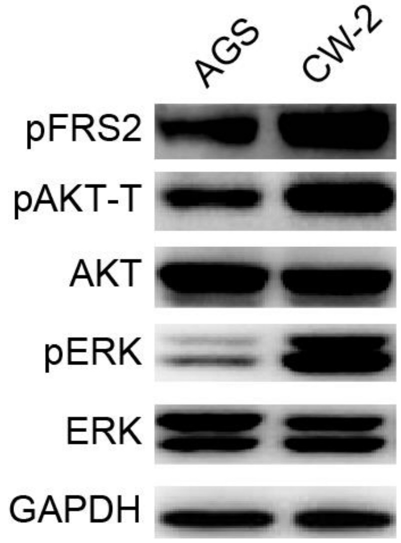

Figure S7: pAKT-T and pERK levels were significantly higher in CW-2 cells compared to AGS WT control cells. Western blot analyses of the indicated proteins were performed in AGS and CW-2 cell lines.

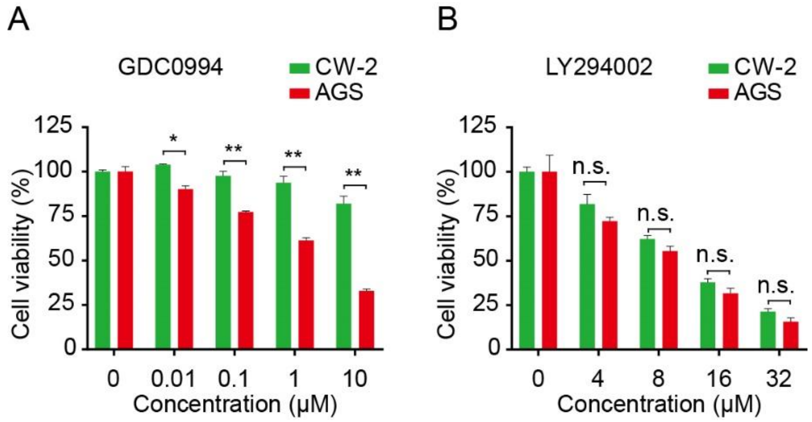

Figure S8: CW-2 cells are more resistant to ERK inhibitor GDC0994 but not to PI3K inhibitor LY294002 compared with AGS cells. (A) Cell viability of CW-2 and AGS cells treated with the ERK inhibitor GDC0994 at the indicated concentrations for 3 days. (B) Cell viability of CW-2 and AGS cells treated with the PI3K inhibitor LY294002 at the indicated concentrations for 3 days. * $P < 0.05$, ** $P < 0.01$, *** $P < 0.001$, and n.s., not significant.

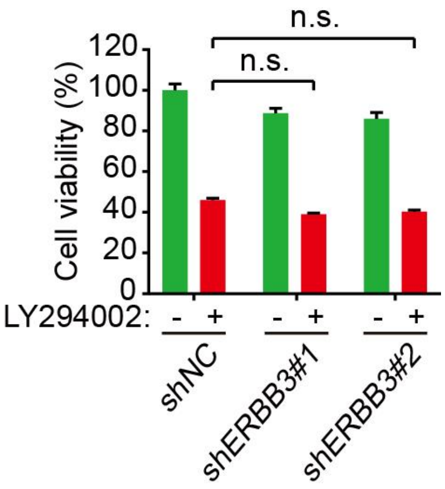

Figure S9: Knocking down of *ERBB3* did not affect sensitivity of CW-2 cells to the treatment of PI3K kinase inhibitor LY294002. Cell viability was measured in CW-2 cells subjected to shRNA-mediated ERBB3 knockdown followed by 3 days of treatment with 4 μM LY294002 * $P < 0.05$, ** $P < 0.01$, *** $P < 0.001$, and n.s., not significant.

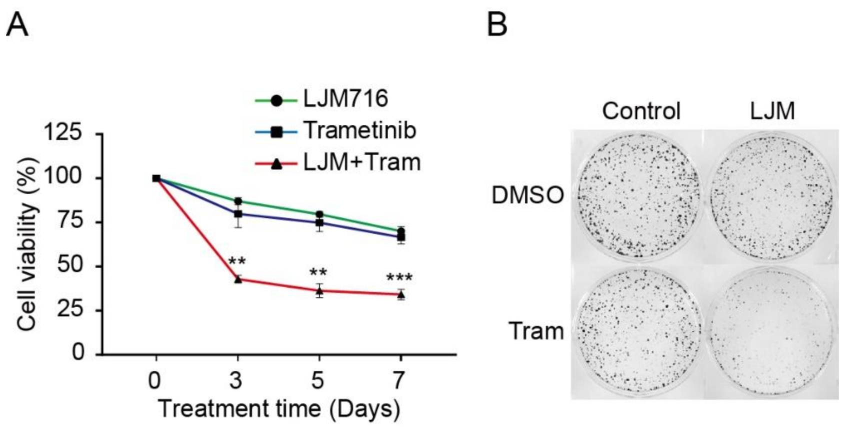

Figure S10: LJM716 and Trametinib (MEK1/2 inhibitor) co-treatment significantly decreased cell viability and reduced foci formation of CW2 cells. (A) Time course of cell viability of CW-2 cells treated with either 10 μg/ml LJM716 alone, 0.01 μM Trametinib alone or both LJM716 and GDC0994. (B) 2 weeks foci formation was measured for CW-2 cells treated with DMSO (control), 10 μg/ml LJM716, 0.01 μM Trametinib or both LJM716 and Trametinib.

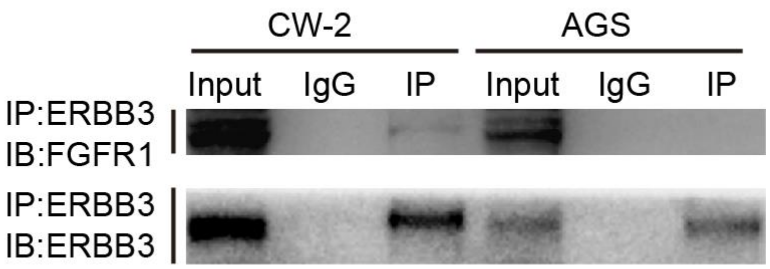

Figure S11: FGFR1 interacts with E928G mutant ERBB3 in CW-2 cells, but not WT ERBB3 in AGS cells. Cell lysates from CW-2 and AGS cells were subjected to Co-IP using anti-ERBB3 antibody.

Supplementary Tables

Supplementary Table 1: Summary of the shRNA sequences used in this study

shRNA	Sequence 5'-3'
shERBB3#1	CCGGTATATGAATCGGCAACGAGATCTCGAGATCTCGTTGCCGATTCATATATTTTTG
shERBB3#2	CCGGAGGTTAGGAGTAGATATTGACTCGAGTCAATATCTACTCCTAACCTCTTTTTG
shFGFR1#1	CCGGCGAGGCATTATTTGACCGGATCTCGAGATCCGGTCAAATAATGCCTCGTTTTT
shFGFR1#2	CCGGCAGAGGAGAAAGAAACAGATACTCGAGTATCTGTTTCTTTCTCCTCTGTTTTT

Supplementary Table 2: Summary of the primers used in this study

Primer	Sequence 5'-3'
ERBB3 forward primer	CCGCTTGACTCAGCTCACC
ERBB3 reverse primer	GTCCCTCCAGTCAATTGTGTCC
FGFR1 forward primer	TCAGGGGAGGATTCCGTCTT
FGFR1 reverse primer	GCGTTTGAGTCCGCCATTG

Materials and methods

Cell culture

Cells in this study including CW-2, KYSE150, HCT15, HCT116, and AGS were purchased from the Cell Bank at the Chinese Academy of Sciences (Shanghai, China). Cells were cultured as instructed. All culture media contained 10% (v/v) fetal bovine serum (FBS), and all cells were cultured at 37°C in 5% CO_2.

TKIs for use in *in vitro* and *in vivo* experiments

Except where indicated otherwise, all TKIs were obtained from Selleck Chemicals. Lapatinib (#S2111), Gefitinib (#S1025), Linsitinib (#S1091), Apatinib (#S5248), Tivantinib (#S2753), BGJ398 (#S2183), PD173074 (#S1264), BLU9931 (#S7819), BLU554 (#S8503), GDC0994 (#S7554), Trametinib (#S2673), LY294002 (#S1105), and LJM716 (Novartis) were dissolved in either DMSO or culture medium at room temperature. For *in vivo* experiments, PD173074 was prepared in 12.5% (v/v) Cremophor EL containing 2.5% (v/v) DMSO.

Cell viability assay

GI cancer cells were seeded in 96-well plates; 24 hours later, the cells were treated with clinically available TKIs at various concentrations for various times. Cell viability was measured using the CellTiter-Glo Luminescent Cell Viability Assay (#G7572, Promega) in accordance with the manufacturer's instructions.

Foci formation assay

Cells (500 cells/well) were incubated in 6-well plates at 37°C for 24 hours, and then treated with drugs and DMSO as control; every 3 days, the culture medium was replaced with fresh medium containing the corresponding drugs. When the cells grew to the level of visible colonies (i.e., foci), the cells were washed once in phosphate-buffered saline (PBS) and fixed in 4% paraformaldehyde (Sigma-Aldrich) for 20 mins. The cells were then stained with 0.5% crystal violet diluted in 20% methanol for another 15 mins at room temperature, and the number of colonies was counted using light microscopy.

Lentivirus shRNA system for gene knockdown

HEK293T cells were co-transfected with the lentiviral pLKO.1 vector (#00456, TranSheepBio, Shanghai, China) containing the shRNA hairpin sequence, the psPAX2.0 packaging plasmid (#TV00772, TranSheepBio), and the pMD2.G envelope plasmid (#TV00457, TranSheepBio). The lentivirus-containing supernatant was collected, concentrated, and used to infect cells, which were cultured for 72 hours. Stably transfected cells were screened using puromycin (#ST551, Beyotime Biotech). The specific shRNA hairpin sequences used to knock down *ERBB3* and *FGFR1* are listed in Supplementary Table 1.

Quantitative real-time PCR

Total RNA was extracted with TRIzol (#3101-100, Pufei Biotech), and the concentration and purity were measured using a spectrophotometer. RNA was reverse-transcribed using the PrimeScript RT Kit (#RR037A, Takara), and quantitative PCR

was then performed using a CFX96 Real-Time System (Bio-Rad) with SYBR Green Supermix (#B21202, Biomake) in accordance with the manufacturer's instructions. The fold difference in gene expression was calculated using the 2-△△Ct method and is presented relative to *GAPDH* mRNA. All reactions were performed in triplicate, and specificity was monitored using melting curve analysis. The PCR primers used in this study are listed in Supplementary Table 2.

Western blot analyses

Total proteins were prepared by homogenizing the cells in RIPA buffer containing protease inhibitors. The homogenate was cleared by centrifugation at 4°C for 15 mins at 12,000 rpm, and the supernatant (containing the protein fraction) was collected. Protein concentration in the supernatant was measured using the BCA Protein Assay Kit (#P0009, Beyotime Biotech), and 30-100 μg of denatured proteins were loaded into each lane and separated on a 10% SDS polyacrylamide gel, and then transferred onto PVDF (polyvinylidene fluoride) membranes (#1620177, Bio-Rad). The membranes were first blocked with 5% (w/v) fat-free milk in Tris-buffered saline containing 0.1% Tween-20, then incubated with primary antibodies at 4°C overnight. The following antibodies were used (except where indicated otherwise, all primary antibodies were from Cell Signaling Technology): pERBB3-Y1289 (1:1000; #4791), ERBB3 (1:1000; #12708), pFRS2-Tyr436 (1:1000; #3861), FGFR1 (1:1000; #ab829), pERK-Thr202/Tyr204 (1:1000; #4370), ERK (1:1000; #4695), pAKT-Thr308 (1:1000; #2965), pAKT-Ser473 (1:1000; #3787), AKT (1:1000; #9272), pJNK-Thr183/Tyr185

(1:1000; #9255), JNK (1:1000; #9252), p-p38-Thr180/Tyr182 (1:1000; #9215), p38 (1:1000; #9212), and GAPDH (1:10000; #MB001, Bioworld Technology). The membranes were then washed and probed with the appropriate horseradish peroxidase–conjugated secondary antibodies (1:3000; #A0216 and #A0208, Beyotime Biotech); finally, the signal was detected using the Pierce ECL System (#32106, Thermo Scientific).

Co-immunoprecipitation (Co-IP)

For Co-IP experiments, the ERBB3-FLAG and FGFR1-MYC expression plasmids were co-transfected into HEK293T cells using a liposomal transfection reagent (#40802ES02, Yeasen Biotech, Shanghai, China). Three days after transfection, whole-cell lysates (1 mg) in cell lysis buffer were incubated with Protein A+G Agarose Beads (#P2012, Beyotime Biotech) and anti-FLAG (1:1000; #AE004, ABclonal, Woburn, MA) or anti-MYC (1:1000; #2276, Cell Signaling Technology) antibody, followed by western blot analysis. For endogenous protein Co-IP experiments, whole-cell lysates from CW-2 and AGS cells were incubated with Protein A+G Agarose Beads (#P2012, Beyotime Biotech) and anti-ERBB3 antibody (#sc-285, SANTA CRUZ,), followed by western blot analysis.

GI cancer nude mouse xenograft model

5-week-old female BALB/c nude mice were purchased from Vital River Laboratory Animal Technology Co. Ltd. (Beijing, China), and maintained under specific pathogen–free conditions at the Laboratory Animal Center of Zhejiang University. All

animal experiments were carried out in accordance with the National Institutes of Health guide for the care and use of laboratory animals. And this study was approved by the Ethic Committee of the Laboratory Animal Center of Zhejiang University. A total of 5×10^6 CW-2 cells were suspended in 100 µl PBS, mixed with 50% Matrigel (#354248, BD Biosciences), and injected subcutaneously into the flanks of the mice. Tumor size was measured using a digital Vernier caliper, and tumor volume (in mm^3) was calculated using the following equation: Tumor volume = (length × $width^2$)/2, with length and width measured in mm. When the tumor size reached 50 mm^3, mice were randomly assigned to four groups of 5 mice each. Every two days, each group received an intraperitoneal injection of vehicle (control), LJM716 (25 mg/kg body weight), PD173074 (20 mg/kg body weight), or both LJM716 (25 mg/kg body weight) and PD173074 (20 mg/kg body weight). The health status of each animal was monitored daily, including any change in weight. After 3 weeks, the mice were euthanized and the xenograft tumors were harvested, photographed, and then fixed in 4% paraformaldehyde and embedded in paraffin for subsequent immunohistochemistry.

Immunohistochemistry

After removal as described above, the tumors were fixed overnight in 4% paraformaldehyde. The fixed tumors were then treated with gradient methanol to remove any air, embedded in paraffin, and sectioned at 4 µm using a rotary microtome. Following paraffin removal and antigen retrieval, the sections were incubated with the following primary antibodies: anti-Ki-67 antibody (5 µg/ml, #14-5698-82, Invitrogen

eBioscience), anti-pERBB3 (1:1000, #4791, Cell Signaling Technology), and anti-pERK (1:400, #4370, Cell Signaling Technology). Staining was visualized using the appropriate HRP-conjugated anti-rabbit or anti-mouse secondary antibody with the REAL EnVision Detection System (#K5007, Dako). The sections were counterstained with hematoxylin. The TdT-DBA In Situ Apoptosis Detection Kit (#4810-30-K, R&D Systems) was also used to stain tumor sections. Photomicrographs were obtained using an Eclipse E400 microscope (Nikon).

Statistical analyses

Data were analyzed and plotted using SPSS 19.0 or GraphPad Prism v6.0 (La Jolla, CA), and all summary data are presented as the mean ± the standard deviation (SD). Two groups were compared using the Student's *t*-test, and multiple groups were compared using a one-way ANOVA followed by Tukey's post hoc test. Differences with a *P*-value <0.05 were considered statistically significant.
